# Tako-Tsubo Cardiomyopathy in a Patient with Advanced Colorectal Adenocarcinoma

**DOI:** 10.1155/2010/487579

**Published:** 2010-06-06

**Authors:** Yusuf Kasirye, Ihab B. Abdalrahman

**Affiliations:** Department of Internal Medicine, Marshfield Clinic, Marshfield, WI 54449, USA

## Abstract

Transient left ventricular dysfunction syndrome (TLVDS), or Tako-Tsubo cardiomyopathy (TC), is a clinical entity in which patients present with features of acute coronary syndrome, electrocardiogram abnormalities, and transient left ventricular (apical or mid-ventricular) dysfunction. Patients usually recover from this condition four to six weeks after the event. The etiology or triggering factors of TC remains unknown. Various triggering factors have been associated with this syndrome, with one of the most recent being malignancies. In this case report we present a postmenopausal female with underlying advanced malignancy who presented with TC. This is consistent with a recent hypothesis that in addition to currently known triggering factors, malignancies might well trigger TC in the context of a stressor or paraneoplastic phenomenon.

## 1. Introduction

Tako-Tsubo cardiomyopathy (TC) is a clinical entity first described in 1991 by Dote et al. [1]. It was initially described as *tako-tsubo,* a word derived from the Japanese octopus trap, which has a similar appearance as the left ventricle during this event. Other names include stress-induced cardiomyopathy, apical ballooning syndrome, ampulla cardiomyopathy, or Broken Heart syndrome. It was first thought to be restricted to the apex of the left ventricle, hence the name “apical”; however, we now have evidence that other parts of the left ventricle, as well as the right ventricle, are involved [2, 3]. Therefore, TC remains the most appropriate name although the most current literature still refers to it as a “left ventricular” or “apical” syndrome.

There continues to be a constant evolution in our understanding of this unique condition as far as etiology, pathophysiology, and triggering factors are concerned. There are no large randomized or cohort studies available. Most of the information known comes from case reports and case series. Current incidence is unknown; however, some studies estimate 1% to 2% of all patients present with acute coronary syndrome, which puts the incidence at 7,000 to 14,000 cases per year. The condition is common in postmenopausal women, with a mean age of 58 to 75 years, and <3% under age 50 [4]. 

Triggering factors and their mechanisms continue to generate deep clinical interest. Recent retrospective studies [5, 6] have unearthed a possible link between malignancies and TC leading to the hypothesis mentioned above. It is in support of this hypothesis that we present this case.

## 2. Case Presentation

A 66-year-old woman with hyperlipidemia and hypertension presented with acute onset of chest pressure. She denied any shortness of breath, diaphoresis, palpitations, presyncopal, or syncopal symptoms. She had no cardiac or diabetic history. She did not have regular medical care. 

Clinically she was in mild distress with tachycardia at 120 bpm. Other vital signs were within normal limits. Physical examination was normal except for positive stool Guaiac test. Laboratory values were troponin I 6.5 ng/mL, creatinine kinase (isoenzyme—MB) 28.4 ng/mL, white blood cell count (WBC) 19600/uL with 0% bands, hemoglobin 10.9 g/dL, hematocrit 32.7%, normal platelets, alanine transaminase 36 U/L, aspartate transaminase 44 U/L, total alkaline phosphatase 234 U/L, sodium 133 mmol/L, and potassium 3.3 mmol/L.

**Figure 1:**
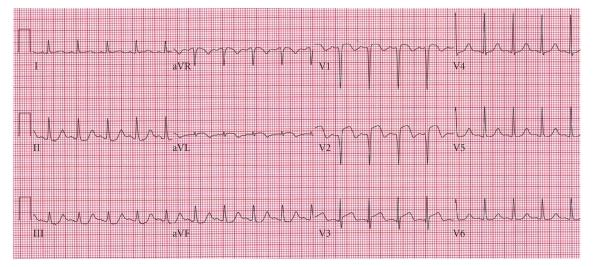
Significant ST segment elevation in precordial leads V1–V3 noted at the time of patient presentation.

**Figure 2:**
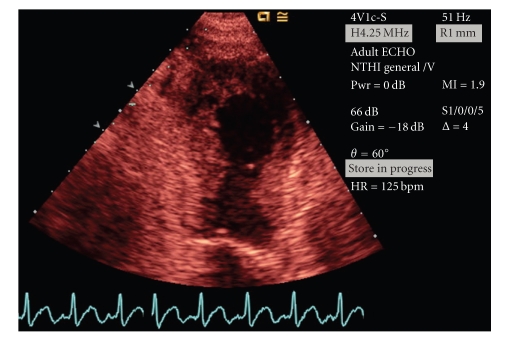
Left ventricular apical akinesia and ballooning visualized during systole on echocardiography.

**Figure 3:**
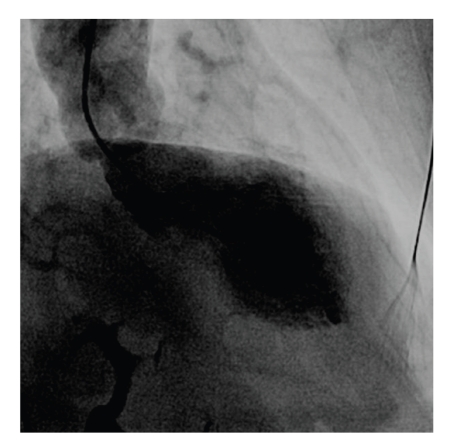
Anteroapical ballooning of left ventricle during systole as seen on left ventriculogram.

Electrocardiogram (ECG) showed ST segment elevation in precordial leads V2-V3 (Figure 1). Chest X-ray was normal. Echocardiography showed apical and anterior wall akinesis (Figure 2). Coronary angiogram revealed normal coronary vasculature. Left ventriculogram showed ejection fraction 36% and anteroapical akinesia with an anteroapical ballooning (Figure 3). A comprehensive viral screen to rule out viral myocarditis as an underlying cause of elevated myocardial enzymes was negative. The patient was managed per acute coronary syndrome protocol and was discharged after two days on carvedilol, lisinopril, and aspirin. The patient denied any psychosocial stressful event prior to presentation.

Because of her positive Guaiac test and mild anemia, she was advised to return in four weeks for a diagnostic colonoscopy. Colonoscopy revealed a colorectal mass with colonic obstruction. Histopathology was consistent with a poorly differentiated adenocarcinoma. Computed tomography (CT) of the abdomen and pelvis revealed stage IV adenocarcinoma for which an exploratory laparotomy with diverting sigmoid colostomy and mucous fistula was performed. This was followed by adjuvant chemotherapy with FOLFOX (folinic acid, fluorouracil, and oxaliplatin) regime. She is currently having a sixth cycle. 

Repeat echocardiography at four weeks postcardiac event showed improved ejection fraction (60%) and resolution of the anteroapical akinesia. Final diagnosis was TC possibly triggered by underlying advanced malignancy.

## 3. Discussion

The pathophysiology of TC remains largely unknown, but various hypotheses have been put forward including, but not limited to, autonomic dysfunction resulting in catecholamine-induced myocardial injury. In 70% of patients there is a triggering factor (psychosocial or physical) identified. Additional hypotheses include catecholamine-induced myocardial stunning, ischemia-mediated stunning due to coronary vasospasm, and myocarditis [7–10]. The predominance in middle-aged females has also raised a possibility of hormonal influence, especially estrogen withdrawal in this age group. A case report of a patient with TC and type 1 CD36 disorder has been described, leading to a suspicion that perhaps there could be a link with abnormal fatty acid metabolism [11]. 

The most supported hypothesis is the catecholamine-induced myocardial stunning, as evidenced by the elevated levels of catecholamine prevalent in patients presenting with TC. Endomyocardial biopsies done in symptomatic patients have shown features of catecholamine toxicity [8]. In this hypothesis, a stressor triggers a catecholamine surge in a susceptible individual, leading to impaired myocardial perfusion, myocyte injury, and rarely left ventricular outflow tract obstruction. The combined molecular effect of these insults on metabolic pathways gives rise to TC [4, 7–10]. This hypothesis is still insufficient to explain issues such as female predominance, age group affected, low recurrence risk, or the failure to reproduce the disease in animal models. 

Recently Burgdorf et al. [5] observed a possible link between malignancies and occurrence of TC. In a retrospective cohort study of 50 TC patients, 18% had a previous history of malignancy, with another 14% developing malignancies over a mean 2.8-year follow-up. Since the occurrence of malignancies was higher in this cohort than the general population, it led them to speculate that TC might be a paraneoplastic phenomenon. Cancers are thought to cause catecholamine-induced myocardial injury through either increasing cardiac sympathetic activity or distorting cardiac adrenoceptor sensitivity. The distortion of adrenoceptor sensitivity might be caused by manipulation of the protein synthesis process intracellularly or extracellularly through possibly adrenoceptor autoantibodies. Burgdorf also speculated that cancer probably lowers the psychic threshold to stress, hence resulting in elevated levels of stress hormones which act on aggravated adrenoceptors [5]. In a larger retrospective study of 100 patients, Elesber et al. [6] found the mortality due to malignancies to be <1% over a mean 4.4-year follow-up period. 

What makes our case more interesting is the close relationship known to exist between adrenoceptor dysregulation and proliferation of adenocarcinoma cells, especially breast and colon [12, 13]. It is possible that during the process of inducing self-proliferation, the cancer cells cause adrenoceptor dysregulation affecting the cardiac cells, resulting in TC. Unfortunately, neither Burgdorf [5] nor Elesber [6] provided the histopathological characteristics of the cancers among their subjects. Even then, this hypothesis might not explain why TC does not occur in all people with adenocarcinoma, which implies that there are other factors at play. 

Most patients present with symptoms of acute coronary syndrome, chest pain, shortness of breath, cardiac enzyme elevation (mild), ST segment elevation, T wave inversion, or QT prolongation. Rare presenting symptoms include syncope, cardiac arrest, cardiogenic shock, or congestive failure. Patients in the intensive care unit might have pulmonary edema. Unique to this entity is preceding emotional or physical stress, although this is absent in about 30% of cases. 

Echocardiography reveals regional wall motion abnormalities. Definitive diagnosis is made on cardiac catheterization, which reveals two classical features: normal coronary arteries and aneurismal dilatation or ballooning of apical segment of the left ventricle (LV), appearing like a Japanese octopus trap on ventriculography. Other variants described are apical sparing or inverted tako-tsubo.

Various diagnostic criteria exist, none of which has been adopted universally. The common criteria used are the proposed Mayo Clinic criteria, initially proposed by Bybee et al. [7] but recently revised by Prasad et al. [4] into the modified Mayo Clinic criteria. These criteria emphasize four characteristics a patient must have in order to be diagnosed with TC. These are transient hypokinesis or apical akinesis not limited to one vascular distribution, absence of significant obstructive coronary lesion, new ECG abnormalities with modest rise in cardiac troponin, and absence of myocarditis or pheochromocytoma. Our patient met the modified Mayo Clinic criteria.

Management is supportive. Since by the time of diagnosis most patients are on treatment for acute coronary syndrome, this is usually continued. The benefit of beta-blockers is unknown although theoretically, since they have an anti-catecholamine effect, they are given, especially those with both alpha- and beta-receptor blockade, like carvedilol. Diuretics are recommended for heart failure. Precautions must be taken to avoid thrombolysis as it might cause harm. Short-term anticoagulation can be given to patients who present with atrial fibrillation or thrombus formation [14]. Aspirin and renin angiotensin system inhibitors are not needed if the patient has no atherosclerosis or recovers systolic function, respectively. 

Complications include left ventricular free-wall rupture, ventricular arrhythmias, mitral regurgitation, left ventricular mural thrombus formation, dynamic intraventricular obstruction, and death [7, 14–19]. In-hospital mortality from TC is estimated at 1% to 2%, with long-term survival the same as a general age-matched population. Hence, prognosis is good, with patients' LV systolic function recovering after four to six weeks, although it can sometimes take up to a year. Recurrence is less than 10% [4, 6].

This case hence raises two other interesting clinical issues. The first issue concerns which level of the troponin I elevation is clinically useful, and whether a degree of elevation matters. In this patient the troponin I elevation was not what we can refer to as modest, yet she did meet the modified Mayo Clinic diagnostic criteria. This challenges the clinical emphasis placed on “modest” troponin I elevation. The second issue is that the studies by Elesber [6] and Burgdorf [5] are relatively small, and the mean age of subjects was 60 to 70 years, an age group with a higher risk of malignancies in the general population [20]. This means that the finding of both malignancy and TC could be circumstantial. Nevertheless, from what we already know about the relationship between adenocarcinoma, stress hormones, and adrenoceptor dysregulation, this hypothesis is legitimate. Retrospectively, measurement of adrenoceptor autoantibodies in our patient would have shed more light on this hypothesis. However, this is not readily available in laboratories, and evidence of a direct causal relationship with Tako-Tsubo cardiomyopathy is still lacking. Further exploration with more studies is required.
